# Association of alcohol consumption with abortion among ever-married reproductive age women in Ethiopia: A multilevel analysis

**DOI:** 10.3389/fgwh.2022.1028166

**Published:** 2022-12-14

**Authors:** Galana Mamo Ayana, Temam Beshir Raru, Alemayehu Deressa, Lemma Demissie Regassa, Mulugeta Gamachu, Belay Negash, Abdi Birhanu, Bedasa Taye Merga

**Affiliations:** ^1^ School of Public Health, College of Health and Medical Science, Haramaya University, Harar, Ethiopia; ^2^ School Medicine, College of Health and Medical Science, Haramaya University, Harar, Ethiopia; ^3^ Department of Public Health, Rift Valley University, Harar, Ethiopia

**Keywords:** abortion, multilevel, consumption, alcohol, regression

## Abstract

**Background:**

A miscarriage or a spontaneous loss of a pregnancy that occurs before the 20th week is an abortion. Even though numerous recommendations state that pregnant women should abstain from alcohol at all stages of pregnancy, alcohol intake among pregnant women is common. However, there are few papers addressing the effect of alcohol use on miscarriage using nationally representative data. Moreover, the association of alcohol use with abortion and its mechanisms is not well studied in the Ethiopian region. Therefore, the objective of the current study was to estimate the association of alcohol use with abortion rates among reproductive age (15–49) women in Ethiopia.

**Methods:**

Using the most recent findings of the Ethiopian Demographic and Health Survey (EDHS), secondary data analysis was performed among pregnant women in Ethiopia. A total of 11,396 women between the ages of 15 and 49 years who were of reproductive age were included in the research. To characterize the study population, descriptive statistics were used. The variability was considered using the multilevel binary logistic regression model. A multilevel binary logistic model was used to determine the effect of alcohol intake on abortion while controlling for potential confounders. In the multivariable analysis, variables with a *P*-value of less than 0.05 were considered statistically significant for the response variable.

**Results:**

The proportion of women who had an abortion was 10.46% with a 95% CI of 9.92–11.03. In the ﬁnal model of the multilevel analysis, age group [adjusted odds ratio (AOR) = 6.13; 95% CI: 3.86–9.73], education level (AOR = 1.29; 95 and CI: 1.10–1.51), alcohol consumption (AOR = 1.38; 95% CI: 1.18–1.61), age at first sex (AOR = 1.20; 95% CI: 1.03–1.39), media exposure (AOR = 1.28, CI: 1.10–1.48), contraceptive use (AOR = 1.34, CI: 1.16–1.56), and occupation of respondent (AOR = 1.21, CI: 1.06–1.38) were identified to be significant determinants of abortion in Ethiopia.

**Conclusion:**

Sexual and reproductive health education and family planning programs should target older women in the reproductive age group, women with primary educational status, working women, and those who initiated sexual intercourse at a younger age considering it could reduce abortion and unintended pregnancy. Furthermore, as part of sexual and reproductive health education, the adverse effect of alcohol consumption on abortion should be emphasized.

## Background

Abortion, often known as a miscarriage or a spontaneous loss of pregnancy, occurs frequently before the 28th week all over the world (1, 2). Alcohol consumption during pregnancy in sub-Saharan Africa (SSA) was high, including in Ethiopia (3), and also alcohol consumption-related pregnancy loss among pregnant women was prevalent (4). The consumption of alcohol among pregnant women has been analyzed, and the potential consumption effects of these substances use majorly increase pregnancy loss among pregnant women (5, 6). Alcohol use during pregnancy is a serious public health issue since it has several detrimental impacts on the mother's and the unborn child's health (7).

There should be targeted interventions, screening programs, and education campaigns for women of childbearing age due to the high prevalence of alcohol consumption and binge drinking during pregnancy in some African nations (8). This is because these behaviors have been linked to a number of negative birth outcomes, including stillbirth, spontaneous abortion, intrauterine growth retardation, low birth weight, preterm birth, congenital malformations, and fetal alcohol spectrum disorder (8–10).

A prospective study conducted in northwest Ethiopia stated that the incidences of low birth weight, pregnancy loss, and stillbirth in Ethiopia were 12.63%, 6.05%, and 4.27%, respectively (11). Additionally, among those who only consume five drinks a week or less, every extra drink was linked to a 6% higher chance of miscarriage (12). In most circumstances, abortion cannot be stopped. However, by leading a healthy lifestyle and avoiding unhealthy risk factors, including smoking, secondhand smoke, alcohol use, and drug use, the odds of a healthy pregnancy may be enhanced and perhaps the risk of miscarriage can be reduced (13, 14). High levels of alcohol intake can result in insulin resistance and a high body mass index (BMI), both of which are risk factors for a higher chance of spontaneous miscarriage (15, 16).

Various studies have revealed that a large proportion of pregnant women still drink alcohol, which is a risk factor for abortion. However, to protect the baby intrauterine from hazards like abortion, they should have to abstain from doing so during their pregnancies (3, 17–19). Due to the fact that many expecting moms, especially those in Ethiopia, have not given enough thought to the consequences of alcohol use during pregnancy, a large percentage of pregnant women drink alcohol (20, 21).

The high prevalence of alcohol use during pregnancy may indicate that all women who are trying to conceive and pregnant women should receive comprehensive interventions and strategies to address the burden of the issue through antenatal care (ANC), reproductive health services, and other mechanisms. One of the few avoidable and modifiable risk factors for unfavorable pregnancy and delivery outcomes is alcohol use during pregnancy. However, Ethiopia lacks sufficient research that examines the effects of alcohol use on miscarriage or spontaneous abortion using nationally representative data. Moreover, the association of alcohol use with abortion and its mechanisms is not well studied in the region. Therefore, this aimed to find out whether alcohol use is associated with abortion in Ethiopia using a multilevel mixed-effects model.

## Methods and Material

### Study setting and data source

Secondary data analysis was conducted based on the 2016 EDHS data. The EDHS is a nationally representative survey conducted in the nine regional states [Afar; Amhara; Benishangul-Gumuz; Gambela; Harari; Oromia; Somali; Southern Nations, Nationalities, and People's Region (SNNPR); and Tigray] and two administrative cities (Addis Ababa and Dire Dawa) of Ethiopia every 5 years (22). The 2007 Population and Housing Census was used as the sampling frame for the EDHS, and a stratified two-stage cluster sampling procedure was used. In the initial stage, 645 enumeration areas (EAs) were chosen independently within each sampling stratum with a probability proportionate to the EA size. In the second stage, 28 households on average were systematically chosen. The full EDHS 2016 report contains detailed information on the sampling process (22).

The demographic and health survey (DHS) program's official database, www.measuredhs.com, was used for the purposes of the current investigation after permission was obtained through an online request that detailed the objectives of the study. From the individual Record (IR file) data set, significant independent and dependent factors were retrieved. The DHS program conducts face-to-face interviews with women of reproductive age (15–49 years) to obtain data on abortion/termination of pregnancy using a single dichotomous (Yes/No) question. The pertinent data were gathered from qualified ever-married women between the ages of 15 and 49 years.

### Population

The source/study population for this study was all reproductive-age (15–49 years) women in the selected enumeration areas within 5 years before the survey in Ethiopia who were ever married. Specific women with no data for the outcome variable and alcohol use were excluded from the study. Accordingly, a total of 15,683 reproductive-age women were found from the 2016 DHS survey. From the total 15,683 reproductive-age women who were not ever married, who had no abortion, and who had alcohol consumption records were excluded from the study. Finally, 11,396 reproductive-age women who met the requirements for inclusion criteria were considered for final analysis.

### Variables of the study

#### Outcome Variables

The study's dependent variable was abortion. The dichotomous answer variable is recorded as 1 if a woman has experienced an abortion and 0 if not.

#### Independent variables

Independent variables of the study were selected by reviewing previously conducted studies. The independent variables were age at first sex, age at marriage, current age, place of residence, marital status, education, occupation, alcohol consumption, cigarette smoking, wealth, region, number of children, contraceptive use, wanted pregnancy, and media exposure. Four statistical models were fitted for these independent variables. The first model was a null model, which was fitted without independent variables. The second model was Model I, which was fitted with individual-level variables (age at first sex, age at marriage, current age, marital status, education, occupation, alcohol consumption, cigarette smoking, wealth, number of children, contraceptive use, wanted pregnancy, and media exposure). The third model was Model II, which was fitted with community-level variables (residence and region). The fourth model was Model III, which was fitted with individual-level and community-level variables.

### Data processing and management

Data were accessed and analyzed using STATA version 14 statistical software. The data were weighted using sampling weight, primary sampling unit, and strata before any statistical analysis to ensure the representativeness of the survey and to take into account the sampling design when calculating standard errors to get reliable statistical estimates.

### Statistical analysis

Descriptive statistics were performed and summarized using tables, graphs, and texts. The frequency and percentage were used for categorical variables, and the mean with standard deviation was used to summarize the continuous variables. The important assumptions such as chi-square and multicollinearity assumptions were checked prior to fitting the statistical model. Since the participants in the same cluster share similar characteristics, the independence of observations and equal variance across clusters’ independence assumption was violated. Therefore, to ensure the reliability of standard errors and unbiased estimates, multilevel advanced statistical modeling is important to overcome the violated independence assumption and to consider the variability between clusters.

The bivariable multilevel mixed-effects logistic regression model was fitted for all independent variables, and the variables with *P*-value 0.20 in the bivariable multilevel mixed-effects logistic regression analysis were candidates for the multivariable two-stage mixed-effects logistic regression. Four different mixed-effects models were fitted (Table 1). A mixed-effects model with the lowest information criteria [Akaike information criteria (AIC) and Bayesian information criteria (BIC)] was selected. Variables with a *P*-value ≤0.05 were declared significant determinants of abortion. An odds ratio with a 95% confidence interval was used to measure the strength of the association. A fixed-effects submodel was used to estimate the association between abortion and explanatory variables. To measure cluster variations, an intra-class correlation coefficient (ICC) with standard deviation was used. In addition, the model comparison was done based on AIC and BIC.

**Table 1:** Basic characteristics of ever-married reproductive-age Women in Ethiopia.

Variables	Weighted frequency	Percentage
Current age (years)		
15–19	731	6.29
20–24	1,903	16.36
25–29	2,611	22.44
30–34	2,249	19.33
35–39	1,866	16.04
40–44	1,266	10.88
45–49	1,006	8.65
Educational status		
No education	7,050	60.60
Primary	3,350	28.80
Secondary	762	6.55
Higher	470	4.04
Alcohol consumption		
No	7,412	63.72
Yes	4,220	36.28
Smoking cigarettes		
No	11,541	99.22
Yes	91	0.78
Media exposure		
No	7,167	61.61
Yes	4,465	38.39
Age at first sex		
<18	7,815	67.19
≥18	3,817	32.81
Age at first union		
Never in union	557	4.79
<18	7,317	62.91
≥18	3,758	32.30
Number of living children		
No living children	1,225	10.53
One living child	1,911	16.43
Two and above	8,496	73.04
Contraceptive use		
User	3,877	33.33
Nonuser	7,755	66.67
Occupation		
Not working	5,675	48.79
Working	5,957	51.21
Wealth index		
Poorest	2,219	19.08
Poorer	2,281	19.61
Middle	2,321	19.95
Richer	2,238	19.24
Richest	2,573	22.12
Place of residence		
Urban	2,099	18.04
Rural	9,533	81.96
Religion		
Orthodox	4,963	42.66
Muslim	3,900	33.52
Protestant	2,498	21.48
Other^a^	272	2.33
Region		
Tigray	847	7.28
Afar	108	0.93
Amhara	2,883	24.78
Oromia	4,427	38.06
Somali	358	3.08
Benishangul	125	1.08
SNNPR	2,308	19.85
Gambela	34	0.29
Harari	29	0.25
Addis Ababa	450	3.87
Dire Dawa	63	0.54

SNNPR, Southern Nation, Nationalities, and People’s Region.

^a^ Others: Catholic, traditional, etc.

### Ethical approval

We obtained the data from the DHS website. It is accessible at http://www.dhsprogram.com after registration. The information gathered was solely for the purpose of conducting a research study. We kept all information private and avoided identifying any specific families or people. The National Research Ethics Review Committee (NRERC) of the Ministry of Science and Technology in Ethiopia and the Ethiopian Health Nutrition and Research Institute (EHNRI) Review Board have given their approval for EDHS.

## Results

### Women's characteristics

A total of 11,632 ever-married reproductive-age women were included in the final analysis. Most of the women (2,611, 22.44%) lie in the age group of 25–29 years. More than one-third (36.28%) of the women consume alcohol, but only 91 (0.78%) smoke cigarettes. Nearly two-thirds (67.19%) of the women had sex below the age of 18 years. Most of the women included were followers of the Orthodox religion (4,963, 42.66%) (Table 1).

### Alcohol consumption

The proportion of women who had abortions was 10.46% with a 95% CI of 9.92–11.03. The highest [35.46% with 95% CI (32.82, 38.19)] and lowest [0.18% with 95% CI (0.05, 0. 67) abortion rates were reported in Oromia and Gambella, respectively. There is a significant association between abortion and the educational status of the mothers. The majority (60.60%) of the participants reported not having a formal education, while 4.04% had higher education. Alcohol consumption was significantly associated with abortion. The odds of abortion were 1.52 [crude odds ratio (COR) = 1.52; 95% CI: 1.30–1.76] times higher among the women who consume alcohol compared to those who did not consume (Table 2).

**Table 2:** Description of background factors associated with abortion and bivariable multilevel mixed-effects logistic regression in COR.

Variables	Abortion		COR (95% CI)
	No	Yes	
Current age (years)			
15–19	747	37	Ref.
20–24	1,865	125	1.60 (1.05–2.42)*
25–29	2,250	219	1.94 (1.31–2.90)**
30–34	1,864	245	2.88 (1.93–4.27)**
35–39	1,605	223	3.46 (2.32–5.14)**
40–44	1,062	199	4.47 (2.98–6.70)**
45–49	797	158	4.97 (3.29–7.49)**
Educational status			
No education	5,826	677	Ref.
Primary	2,858	350	0.99 (0.86–1.14)
Secondary	949	107	0.61 (0.45–0.81)**
Higher	557	72	0.74 (0.52–1.05)
Place of residence			
Urban	2,770	408	Ref.
Rural	7,420	798	0.78 (0.633–0.96)*
Region			
Tigray	735	111	0.71 (0.47–1.04)
Afar	96	12	0.53 (0.26–1.08)
Amhara	2,568	314	0.54 (0.38–0.76)**
Oromia	3,995	431	0.48 (0.34–0.677)**
Somali	326	32	0.44 (0.27–0.72)**
Benishangul	118	7	0.25 (0.11–0.58)
SNNPR	2,092	217	0.45 (0.32–0.64)
Gambela	28	6	0.30 (0.07–1.24)
Harari	22	7	0.42 (0.10–1.62)
Addis Ababa	370	80	Ref.
Dire Dawa	55	8	0.64 (0.28–1.48)
Alcohol consumption			
No	6,948	721	Ref.
Yes	3,242	485	1.52 (1.30–1.76)**
Media exposure			
No	6,071	614	Ref.
Yes	4,119	592	1.25 (1.09–1.43)**
Age at first sex			
<18	6,564	827	1.24 (1.08–1.42)**
≥18	3,626	379	Ref.
Number of living children			
No living children	1,237	129	0.60 (0.47–0.76)**
One living child	1,828	176	0.72 (0.60–0.86)**
Two and above	7,125	901	Ref.
Contraceptive use			
User	2,877	302	Ref.
Nonuser	7,313	904	1.43 (1.24–1.65)**
Occupation			
Not working	5,258	534	Ref.
Working	4,932	972	1.31 (1.15–1.49)**

COR, crude odds ratio; SNNPR, Southern Nation, Nationalities, and People’s Region.

*P < 0.05; **P < 0.01.

### Determinants of abortion

#### Random effects

The null model's findings indicated no statistically significant variation in the odds of abortion, with a community variance of 0.35. The null model's ICC also indicated that only 9.62% of the total variation in the odds of abortion could be attributed to differences between communities, indicating that there are no cluster differences. Community variance in the individual-level model (model corrected for individual-level characteristics) was 0.34; an SE of 0.05 was still nonsignificant but decreased, and 9.40% of the overall variation of abortion might be attributed to the community, which still means that there are no cluster differences (Table 3).

**Table 3:** Multivariable multilevel mixed-effects logistic regression analysis of factors associated with abortion among ever-married reproductive-age women in Ethiopia

Variables	Models			
	Null model AOR (95% CI)	Model I AOR (95% CI)	Model II AOR (95% CI)	Model III AOR (95% CI)
Current age (years)				
15–19		Ref.		Ref.
20–24		1.85 (1.20–2.86)**		1.86 (1.20–2.86)**
25–29		2.46 (1.59–3.82)**		2.45 (1.58–3.80)**
30–34		3.65 (2.34–5.69)**		3.57 (2.28–5.58)**
35–39		4.44 (2.83–6.95)**		4.34 (2.76–6.82)**
40–44		5.71 (3.62–8.99)**		5.55 (3.51–8.78)**
45–49		6.13 (3.86–9.73)**		5.98 (3.75–9.52)**
Educational status				
No education		Ref.		Ref.
Primary		1.29 (1.10–1.51)**		1.26 (1.08–1.48)**
Secondary		0.77 (0.56–1.06)		0.72 (0.52–1.01)
Higher		0.82 (0.56–1.21)		0.77 (0.52–1.47)
Alcohol consumption				
No		Ref.		Ref.
Yes		1.38 (1.18–1.61)**		1.33 (1.11–1.59)**
Media exposure				
No		Ref.		Ref.
Yes		1.32 (1.14–1.53)**		1.28 (1.10–1.48)**
Age at first sex				
<18		1.20 (1.03–1.39)*		1.21 (1.04–1.41)*
≥18		Ref.		Ref.
Number of living children				
No living children		1.10 (0.83–1.46)		1.09 (0.82–1.45)
One living child		1.22 (0.99–1.51)		1.19 (0.96–1.48)
Two and above		Ref.		Ref.
Contraceptive use				
User		Ref.		Ref.
Nonuser		1.35 (1.17–1.56)**		1.34 (1.16–1.56)**
Occupation				
Not working		Ref.		Ref.
Working		1.20 (1.05–1.38)**		1.21 (1.06–1.38)**
Place of residence				
Urban			Ref.	Ref.
Rural			1.04 (0.80–1.34)	1.08 (0.82–1.43)
Region				
Tigray			0.68 (0.44–1.06)	0.68 (0.44–1.06)
Afar			0.52 (0.25–1.08)	0.75 (0.35–1.60)
Amhara			0.53 (0.35–0.79)**	0.54 (0.35–0.82)**
Oromia			0.46 (0.31–0.70)**	0.57 (0.37–0.87)**
Somali			0.43 (0.25–0.73)**	0.62 (0.35–1.10)
Benishangul			0.24 (0.10–0.58)**	0.28 (0.11–0.67)**
SNNPR			0.44 (0.29–0.67)**	0.55 (0.35–0.85)**
Gambela			0.29 (0.10–1.22)	0.36 (0.08–1.56)
Harari			0.41 (0.11–1.60)	0.52 (0.13–2.06)
Addis Ababa			Ref.	Ref.
Dire Dawa			0.63 (0.27–1.47)	0.77 (0.33–1.82)
Random effects				
Community variance	0.35 (0.06)	0.34 (0.06)	0.32 (0.05)	0.33 (0.05)
ICC%	9.62%	9.40%	8.94%	9.10%
PCV				
MOR	1.75	1.74	1.71	1.73
Model comparison				
AIC	7,668.88	**7,429.95**	7,660.13	7,435.88
BIC	7,683.56	**7,562.10**	7,755.57	7,648.77

AOR, adjusted odds ratio; SNNPR, Southern Nation, Nationalities, and People’s Region; AIC, Akaike information criteria; BIC, Bayesian information criteria; PCV, proportional change in variance; MOR, median odds ratio.

*P < 0.05; **P < 0.01.

#### Fixed effects

The interpretation of the fixed effects was based on the parsimonious model, which was determined to have lesser AIC and BIC. Compared to previous models, Model I was modified for only individual-level components with minimal AIC and BIC, and this model successfully fitted the data. Age, education, alcohol usage, media exposure, contraceptive use, age at first sex, and employment of the respondent were significant predictors of abortion in Ethiopia in the multivariable analysis at a 5% level of significance.

The odds of abortion were 6.13 [adjusted odds ratio (AOR) = 6.13; 95% CI: 3.86–9.73] times higher among the women in the age group of 45–49 years compared to the women in the age group of 15–19 years. The women who had primary education were 1.29 times more likely to have an abortion compared to those with no formal education (AOR = 1.29; 95 and CI: 1.10–1.51). Women who consume alcohol were 1.38 times more likely to have an abortion compared to their counterparts (AOR = 1.38; 95% CI: 1.18–1.61). Women who had their first sex below the age of 18 years were 1.20 times more likely to have an abortion compared to those who had their first sex at the age of 18 years and above (AOR = 1.20; 95% CI: 1.03–1.39) (Table 3).

## Discussion

The current study depicts the association between alcohol consumption and abortion among ever-married reproductive-age women in Ethiopia. The odds of abortion among ever-married reproductive-age women who consume alcohol were higher compared to those who had no history of alcohol consumption. This is consistent with the available evidence on the relationship between alcohol and risky sexual behaviors. A narrative review conducted in sub-Saharan Africa concluded that alcohol was strongly associated with the increased practice of unsafe sexual intercourse including inconsistent condom use (23). The increased odds of abortion among alcohol consumers might be related to the associated risk of unprotected sex that results in unintended pregnancy and abortion.

Compared to their younger peers (15–19 years), older women (20–49 years) had a greater abortion rate. The results are consistent with the Nigerian DHS, which found that young adults aged 20–24 years had higher odds of abortion than those aged 15–19 years (24). Another study from Ethiopia also indicated that older age is associated with higher odds of abortion compared to younger women (25). The possible explanation might be the increased risk of medical and pregnancy-related complications that might make the pregnancy more difficult and raise the likelihood of an unfavorable pregnancy result, such as abortion (26). Furthermore, chromosomal abnormality risk rises with maternal age and uterine and hormonal function declines, all of which ultimately lead to miscarriage or abortion if women become pregnant later in life (27).

Compared to women who had not been exposed to the media, the likelihood of abortion was higher among those who had media exposure. This result is consistent with research from Ethiopia (25) and Ghana (28). This might be related to the access to information about the availability of abortion services (29). Moreover, women who have media exposure might know available laws related to abortion and be less stigmatized (30).

Educational status was identified as an independent determinant of abortion among ever-married reproductive-age women. Women who attended primary education had higher odds of abortion than women who had no formal education. This finding is supported by evidence from a study conducted in India (31). It could be due to the reason that educated women might have information about the consequence of abortion on their health and access to abortion services (29). As shown in this study, the odds of abortion increased among working women compared to those not working. Other previous studies revealed a significant relationship between abortion and higher socioeconomic status including working status (32, 33). This might be because working women may have the ability to cover the cost of maternal healthcare services. In addition, unemployed women may face cost barriers like transportation costs that could hinder women from having an abortion.

The current study pinpointed the association between contraceptive use and abortion among ever-married reproductive-age women. The odds of abortion were higher among contraceptive nonusers compared to those who were contraceptive users. This might be related to higher rates of unintended pregnancy among contraceptive nonusers that result in abortion (34, 35). Early sexual debut at younger than 18 years was positively associated with abortion. Previous studies also indicated that early initiation of sexual intercourse increased the rate of unintended pregnancy and abortion (36). This study provided evidence of the association between alcohol consumption and abortion among ever-married reproductive women. Moreover, factors such as older age, media exposure, primary education, contraceptive use, age at first sex, and working status have shown an association with abortion. Therefore, the current study recommends sexual and reproductive health education and family planning programs targeting older women, women with primary educational status, working women, and those who initiated sexual intercourse at a younger age to reduce abortion and unintended pregnancy. Furthermore, as part of sexual and reproductive health education, the adverse effect of alcohol consumption on pregnancy outcomes should be emphasized.

## Strengths and limitations of the study

The study has certain limitations. The cross-sectional nature of the data made it difficult to infer any conclusions concerning causality. Alcohol consumption's effects on other variables and spontaneous abortion could only be detected in this study, not their causal relationships. There might be recollection bias as the study relied on reproductive women's reports. There were some possible confounders, such as antenatal anxiety or depression and the quantity of alcohol consumed, that were not considered in the analysis due to the unavailability of data. The current study's conclusions were based on data that were nationally representative despite these limitations. As a result, they provide information that policymakers and programs used to reduce pregnancy loss in Ethiopia.

## Conclusions

This study provided evidence of the association between alcohol consumption and abortion among ever-married reproductive-age women in Ethiopia. Moreover, factors such as older age, media exposure, primary education, contraceptive use, age at first sex, and working status have significant associations with abortion among ever-married reproductive-age women in Ethiopia. Sexual and reproductive health education and family planning programs should target older women, women with primary educational status, working women, and those who have initiated sexual intercourse at a younger age to reduce abortion and unintended pregnancy. Furthermore, as part of sexual and reproductive health education, the adverse effect of alcohol consumption on pregnancy outcomes should be emphasized.

## Data Availability

The raw data supporting the conclusions of this article will be made available by the authors, without undue reservation.
